# The Microbe of Youth

**Published:** 1912-08

**Authors:** 


### EDITORIAL DEPARTMENT.

DR. F. E. DANIEL, Editor    DR. R. H. L. BIBB, Associate Editor

### "The Microbe of Youth."

PROFESSOR METCHNIKOFF'S LATEST DISCOVERY.

Metchnikoff says that a man should live a hundred years; and that he does not do so is due to a cause which he has set himself to find and remove. He says that when a man has lived out his natural term of years the impulse of death cames on as naturally as does that of sleep, and one yields to it without fear or regret, as he does when he lays himself down to sleep. Of course, this is barring accidents; but it seems to me that life, especially that of babies, is so beset with dangers that it is a wonder that anyone ever lives to get grown—let alone—a hundred years.

His latest discovery is that man poisons himself by his food, even the best and most wholesome and nutritious of which, he says, generates within the intestines certain poisons (auto-infection), which he calls the "aromatic series of the intestinal flora"—"indols" and "phenols."

Eggs, for instance, usually regarded as the best and most wholesome diet, "gives the maximum of these aromatic poisons."

To prevent this poisoning of one's self by his diet, or, failing this, to neutralize or antidote these poisons in the intestinal tract, is the problem to be solved, so that man may live out his hundred years. While the first, in all human probability, can never be accomplished—man being an omnivorous animal—it is not so unreasonable to believe that the latter may be possible in some cases.

To this end, Metchnikoff and his assistants in the Pasteur Institute at Paris, of which he is director, performed with amazing painstaking a series of experiments, the result of which, speaking broadly, is that *sour milk* furnishes the antitoxin to those aromatic poisons generated, even by a vegetable diet, and the "microbe of youth" consists of the bacteria that turn the milk sour, and which he has named *Bacillus lactis bulgaricus*.

* * * "They established the fact that indol administered in small doses to rabbits, guinea pigs, and macacos, produced organic lesions, remarkably similar to the lesions observed in old age, in the vascular system, the kidneys, the liver, and the brain. Metchnikoff and Wollman conclude, therefore, that senility is caused largely by poisons of the intestinal flora, notably by the aromatic series, indols and phenols. A natural question was, how could the formation of these poisons be prevented?"

It was found that lactic acid furnished the key to the problem—as stated.

We may soon expect to see the *bacillus lactis bulgaricus* on the market in capsules, "put up expressly for family use." Meantime, however, he recommends buttermilk. That's a very pleasant remedy—at any rate, this hot weather.

The foregoing reflections were generated by reading in the *New York Medical Journal* (July 13th) a synopsis of a letter from Professor Metchnikoff to the editor of that *Journal,* Dr. Sajous, giving a detailed account of the experiments. They were made on white rats, dogs and guinea pigs. The letter was accompanied by a copy of a paper written by the professor in collaboration with his colleague, Eugene Wollman, read before the Paris Academy of Science, June 10, 1912, entitled "Experiments on Intestinal Disinfection."

The importance of the discovery is far-reaching, and constitutes a most valuable contribution to the subject to which Dr. Sajous (the editor of the *New York Medical Journal*) has given so much time and study; his two-volume work on "Internal Secretions" is standard, and a classic.

It is not to be hoped that the *lactis bulgaricus* will prove to be the elixir of life or a specific for indigestion and intestinal intoxication, but it will doubtless prove of benefit in the avoidance or treatment of those obscure continued fevers some have been accustomed to regard as neither typhoid nor malarial, but a little of both (?) and which are vaguely described in the books as *æstivo-autumnale*—a term that means "summer and fall," and which serves as well as any other to conceal ignorance.

Here we have it at last. We eat too much meat,—and other proteids. "It is food of an animal nature," says the professor, "that furnishes most aromatic poisons, the maximum being given by meat and eggs, the white of eggs especially," while "beets and dates act in an opposite manner by furnishing sugar. As it is not the sugars themselves, but the acids produced at their expense, that prevent intestinal infection, there was added to the rat food pure cultures of *bacillus lactis bulgaricus,* which yields lactic acid in large quantities. Thus modified, the regimen finally suppressed all urinary poisons of the aromatic series."

So, you see, my beloved, it is meat-eating that makes a fellow old. You can head off old Father Time, and have a hundred birthdays, if you will live right, and quit drinking whisky—and eating beef—and eggs!

